# Use of a qualitative case study to learn lessons from severe preeclampsia causing a maternal near-miss: a case report

**DOI:** 10.1186/s13256-018-1821-x

**Published:** 2018-09-26

**Authors:** Moti Tolera, Alula M. Teklu, Abdurahman Ahmed, Abdiwahab Hashi, Lemessa Oljira, Zerihun Abebe, Wondimagegn Gezahegn, Kahasse Gebre Kidan

**Affiliations:** 1 Haramaya University School of Public health, Dire Dawa, Ethiopia; 2 MERQ Consultancy, Addis Ababa, Ethiopia; 3 Debre Berhan University, Debre Birhan, Ethiopia; 4 Jigjiga University, Jigjiga, Ethiopia; 5 St. Paul’s Hospital Millennium Medical College, Addis Ababa, Ethiopia

**Keywords:** Maternal near-miss, Maternal complication

## Abstract

**Background:**

Maternal mortality is a critical indicator in assessing the quality of services provided by a health care system. Approximately 99% of all maternal deaths occur in developing countries; where a majority of the causes of these deaths are preventable.

**Case presentation:**

A 25-year-old, married, multigravida, black woman who has had six live births presented to a health center with the chief compliant of abnormal body swelling of 2 days’ duration and loss of consciousness. On arrival to the first contact health center her blood pressure was 170/105 mmHg and her temperature was 36.5 °C. She had generalized swelling, a history of blurred vision, and headache. She had no history of abortion, stillbirth, and cesarean section and no history of antenatal care follow-up. She gave birth to her previous children at home with no history of obstetric complications. The gestational age at the time of arrival was 37 weeks. She was referred to a general hospital for further management.

At the general hospital she was diagnosed as having severe preeclampsia and she was managed with magnesium sulfate and an antihypertensive medication for 2 days. She was counseled to have induction of labor by the attending physician but refused to give consent and went home. She returned to the referral hospital 2 days later after labor had started spontaneously at home and the delivery was a spontaneous vaginal delivery with outcome of a live male baby, his Apgar score was 6/10 immediately after birth and he weighed 1.9 kg.

**Conclusions:**

If there were no previous obstetric problems, the women perceived that she will not face complications in her future pregnancies and stay home until she had developed life-threatening complications. If women visit health facilities and if the health care providers are responsive and there is robust referral in place, maternal and fetal complications will be prevented.

## Background

Maternal complications and mortality is a critical indicator to assess the quality of services provided by a health care systems [1]. A total of 10 million women worldwide are estimated to experience severe complications of pregnancy every year, with half a million of these dying as a result; out of all maternal deaths, 99% occur in developing countries including Ethiopia [2]. Even though some efforts were made by governments of Ethiopia to promote institutional deliveries, births continue to occur in the home settings [3–6]. Ethiopia is the fourth largest contributor country to global maternal deaths, next to India, Nigeria, and the Democratic Republic of Congo [7].

A maternal near-miss case is defined as “a woman who nearly died but survived a complication that occurred during pregnancy, childbirth or within 42 days of termination of pregnancy” [8] and is used for monitoring the implementation of critical interventions in maternal health care. So, a systematic process should be in place to assess the quality of care with an assumption that all maternal deaths involve at least one life-threatening condition (organ dysfunction) [8, 9]. Complications during pregnancy and childbirth can occur at any point of time; therefore, it is important to ensure readiness in terms of infrastructure, human resources, and equipment for timely management of complications at all basic and emergency obstetric care health facilities [1]. Direct obstetric complications account for 85% of maternal deaths as well as many acute and chronic illnesses [10, 11]. Preeclampsia-eclampsia disorder is one of the direct pregnancy-related complications and is a pregnancy-specific hypertensive disorder affecting multiple systems and contribute  a maternal mortality rate of 1.8% [12, 13]. Eclampsia is defined as the occurrence of one or more convulsions superimposed on preeclampsia [4]. Preeclampsia is pregnancy-induced hypertension in association with proteinuria (> 0.3 g in 24 hours) ± edema and virtually any organ system may be affected. Severe preeclampsia is variously defined [1, 4]. There is consensus that severe hypertension is confirmed with a diastolic blood pressure (BP) ≥ 110 mmHg on two occasions or systolic BP ≥ 170 mmHg on two occasions or significant proteinuria (at least 1 g/liter) [14]. Risk factors for preeclampsia include either a very young or advanced maternal age, multi-fetal pregnancy, hemolytic disease of the newborn, diabetes mellitus, chronic systemic hypertension, and renal disease [15]. In developing countries the incidence of eclampsia/preeclampsia poses a great problem in the field of obstetrics due to poor socioeconomic conditions and lack of antenatal care (ANC) follow-up [16]. Hence the aim of this case report was to explore a case of maternal near-miss from the woman’s home to her place of delivery and to share the lessons learnt from this case using a qualitative case study design, which is a rarely reported technique.

## Case presentation

### Materials and methods

The case study was conducted in one of general hospitals in the Somali regional state of Ethiopia. The hospital was established approximately 50 years ago. The study was conducted from February 1 to March 1, 2017; using an explorative qualitative case study design. The tools used to collect data were in-depth interviews (IDIs), key informant interviews (KIIs), and facility abstraction using  World Health Organization (WHO) near-miss assessment tools [8]. Participants of this study were the mother herself, her sister, her male partner, maternal and child health (MCH) coordinators at the health facilities she has visited, the head of the health center/hospital, and the health service providers who assisted her both at first contact and at the referral hospital, such as the gynecologist, midwife, and general practitioner (GP). Purposive sampling was used to select the case as well the participants. A near-miss case was identified from among women with pregnancy-related complications whose diagnosis met the WHO near-miss criteria [8] and who were admitted to the obstetric unit of the hospital. Investigations were conducted for abnormalities, septicemia, anemia, and other organ dysfunction/failure. Data were collected for determining the nature of the obstetric complication, presence of organ system dysfunction/failure, and timing of near-miss events with respect to admission. Fetal outcome and Intensive Care Unit (ICU) admissions were also noted. Detailed information on maternal complication for the underlying cause and time period was obtained. The information was documented using the narrative qualitative method. For this, multiple interviews were carried out. The data collectors had many years of experience with qualitative case study data collection, verbatim transcription, and translation. The data collectors in the field transcribed audio-digital recordings into the local language and then translated the data into English. The data collectors also kept notes and kept records of field reports, field notes, completed questionnaires, and interview recordings. The inductive qualitative data analysis method was used to analyze the data collected. The data were entered, coded, categorized, and analyzed using NVIVO version 11 software. Consent for data collection:

- ◈ Ethical clearance for study was obtained from SPHMMC IRRB and local IRRB Ethio-Somali Regional Health Bureau (ESRHB).
- ◈ Official letters were taken to zonal health departments, Woreda Health Offices, health centers, and health post/kebele (neighborhood) administrators.
- ◈ The study participants were informed about the purpose of the study and written and informed verbal consent was taken.
- ◈ All participants’ right to self-determination was respected.
- ◈ The confidentiality and the privacy of the respondents were maintained.
- ◈ Written informed consent was obtained from the patient for publication of this case report and any accompanying images.

### Patient information

The case was a 25-year-old married, multigravida, black woman. She was gravida 7 and para 6, all surviving. She lived in a rural pastoral area. She had no formal education and her livelihood was pastoralist. She was categorized into a low socioeconomic class. She had no history of abortion, stillbirth, cesarean section (C-section), or any obstetric complications.

### History of current pregnancy

The current pregnancy was her seventh with a gestational age of approximately 37 weeks at presentation. The mother explained as her gestational age increased she started to develop generalized body swelling that started from her eyes, then face, and eventually migrated to her legs. She had history of blurred vision and headache. After two to three episodes of seizure, she was unconscious, and then she was taken to a nearby health facility (Health center) by her husband and mother.

*“I was unconscious when my husband and my mother took me to the nearby health center.”* **The mother**

At the first contact health center her axial temperature was 36.5 °C and her BP was 170/105 mmHg in supine position; she had three episodes of seizure and other clinical findings and no tests were found on the patient's chart.

After this episode, there was no altered state of consciousness and no seizure. She did not have, headache, nausea, visual disturbances, or fever; she had no history of head injury, central nervous infection, no history of alcohol intake, no history of seizure or epilepsy, and she was negative for stigmata of neurocutaneous syndrome.

Because of shortage of supply and instruments, except urine analysis (protein 3+), no other laboratory or other investigative tests were done at the health center. The health care providers at the primary health facility diagnosed her as having preeclampsia and referred her to the nearby general hospital with an ambulance.

### Maternal condition on arrival at the general hospital

The hospital is named as a general hospital but is serving as a referral hospital in the region, providing a wide range of services including approximately 25 deliveries per day.

*“It is serving beyond its capacity. If you look around the maternity area, many women were in labor there; since there is no place we can refer to, we don’t have a limit on admission, and we accept everyone. When you provide service beyond capacity, there will be quality problems. The delivery rate is extremely high in our situation. Sometimes there are up to 25 deliveries per day.”* **Hospital officer**

The mother was visited by GPs, who were assigned to the emergency room at the hospital, as soon as she arrived. The family reported that they brought her to the health facility because she was sick due to an evil spirit and they had unsuccessfully tried spiritual treatment at their locality. Her vital signs on arrival at the general hospital were: BP 180/110 mmHg, temperature 37.6 °C, respiratory rate (RR) 22/minute, and her pulse rate (PR) was 82/minute. The health care providers counseled her family that they can use both modern and spiritual treatments in the hospital and then her family gave consent for admission. Then after the health care providers gave lifesaving emergency treatment using antihypertensive and anti-seizure medications, she was admitted to the obstetrics ward and she underwent full investigations including an ultrasound and laboratory investigations: urine analysis, complete blood count (CBC), and organ function tests like liver function test (LFT) and renal function test (RFT). The laboratory results showed 3+ protein for urine analysis dipstick test which is equivalent to 3 gm/l protein [14] and CBC was in normal range except for hemoglobin and platelets of 7 mg/dl and 100,000/mm^3^, respectively. Her aspartate aminotransferase (AST) result was raised (85 mg/dl) but the RFT result showed no increment in creatinine (1.5 mg/dl). She was finally diagnosed as having severe preeclampsia and full-term pregnancy (even though the diagnosis seemed eclampsia). The pregnancy type was singleton and in the cephalic position as reported by the radiologist. She was started on magnesium sulfate and an antihypertensive treatment (methyldopa). After her BP was controlled and she looked well, she was counseled for induction of labor due to the indication of (preeclamsia/) eclampsia. However, she refused the induction and went home; unfortunately, 2 days later she returned to the hospital soon after labor had started spontaneously at home. She was again admitted to the hospital for delivery. During this time she had no history of loss of consciousness, no history of seizure, no history of blurred vision, and no abnormal findings were detected. Because of abscence of  cardiotocography (CTG) fetal heart monitoring was not done. The mother’s BP was 135/95 mmHg, PR was 80 beats/minute, and respiratory rate (RR) was 25/minute. She had pedal and periorbital edema. After 6 hours follow-up at labor and delivery ward, she gave birth to a live male baby with an Apgar score of 6/10 at 5 minutes after delivery and a weight of 1900 mg. The baby was admitted to the under-5 ward due to having a low birth weight (1.9Kg). She went home with orally administered methyldopa 250 mg to be taken twice a day.

*“After referral to this facility, she was clerked at emergency ward by a general practitioner and then she was admitted and seen by the gynecologist. Then she had full investigations including an ultrasound and she was counseled for induction of delivery. However, she refused induction and preferred to go back home. Then she delivered spontaneously... The fetal outcome was a live but underweight male child... Now it is 24 hours since she delivered and the maternal status is good. ....We will transfer her to her baby in the ICU after we control her blood pressure.”* **MCH focal person of the hospital**

The mother and her family described her condition during the pregnancy as very risky and she thinks she would have been died if she had not gone to the health facility and received treatment.

*“…this was a very risky pregnancy, she was near to death, if she had no intervention here, and she was near to death...”* **The mother’s sister**

*“...after this pregnancy I decided to go to the health facility”* **The mother**

She gave birth to all her six previous babies at home without any problems and was supported by a non-trained traditional birth attendant (n-TBA) because she believed visiting a health facility while healthy was not important during pregnancy. She had no history of ANC attendance for the current pregnancy as she felt well and had no previous complications.

*“Truly speaking I did not attended any ANC for the benefit of my health and that of the fetus but if I get sick I will go to the health facility to get treatment…I had all my previous deliveries in my home without any complication, I think it is ok to deliver in my home when I have no health problems.”* **The mother**

She believed that giving birth at a health institution is only necessary when there are problems during pregnancy that is why she presented to this health facility after her family perceived she had developed some complications.

*“…it is very good to deliver in a health facility when you have a health concern. In my last delivery I went to the health center, all my body was swollen, to get treatment I went there.”* **The mother**

The mother perceived that culture and religion are supportive of seeking health services when needed and religion does not prohibit her from seeking any health services except being attended to by male health care providers and “strange individuals,” meaning individuals who do not know her local language; she perceived the patriarchal system in her community affected her use of the health services she needed as men were the source of finance and were the decision makers.

*“… but it is difficult to get delivery service from males and strange people.”* **The mother**

*“Husband is the source of income for the health care expenses and the family needs husbands who have a good attitude about modern health care utilization, so it is easy for the mother to use.”* **The husband**

The MCH focal person stated that cases of eclampsia and preeclampsia were increasing in this region, and as the cause is unknown it needs further research.

*“The cause of preeclampsia and eclampsia is unknown but cases are increasing in our region. It may be associated with their eating habits. One doctor is doing research into preeclampsia and eclampsia cases to investigate the cause of these problems*.” **MCH focal person of the hospital**

The mother was asked about her personal feelings toward the health facility she was in and the health care providers caring for her; she was satisfied with the services the health care providers provided her but complained that the hospital had inadequate room for labor and childbirth.

*“The health care providers were welcoming and friendly to the patients but in the hospital there are no adequate rooms for the women delivering.”* **The mother**

The MCH focal person also discussed lack of an ICU for delivery. They also do not have enough space for delivery services and the delivery room is very congested. Women were delivering on the floor outside the room.

*“It is known that we don’t have ICU services and this is a critical problem in our set up. I and the gynecologists tried a lot but still there is no solution. There are many problems with this but the main reason is that there is no space for establishing it. As you see the delivery ward is crowded, women are delivering on the floor and there is not even a little space.*” **MCH focal person**

The health care providers complained about the accessibility of ambulances; they reported that most of the ambulances which were provided by the Ministry of Health were assigned for other duties, rather than serving emergency health services or laboring mothers.

*“…This may be the most common complaint related to transportation. It is thought that there should be an ambulance service for all mothers and the Ministry of Health distributed ambulances to all districts even though they are used for other purposes.”* **MCH focal person**

The mother also described the quality of services starting from the kebele (neighborhood) she is living in, to the referral facility where she was treated and gave birth. The health facility near her has a shortage of human resources and supplies including medications, but at the referral health facility, she felt that it had enough human resources and they were serving the admitted patients day and night.

*“….in our kebele the health center does not have enough health care providers and drugs. But it was okay in the hospital as health care providers were with us day and night.”* **The mother**

The MCH focal person reported that the mother preferred the death of her child to a surgical intervention done to save the life of herself and her baby. The intention of the mother was to save her own life rather than saving her child, because it was expected that the mother could give birth to many children if she survived. A lot of time was spent counseling the mother to have a C-section and other interventions. Even the father’s focus was not on the baby but on the life of the mother.

*“First, the community in our region doesn’t worry about their children. Even the mothers themselves don’t worry about their beloved child. A mother will prefer the death of her fetus rather than surgical delivery. They fear complications of operations and consider it as if we are going to slaughter them. One doctor is doing research on how long it takes to counsel and get consent for cesarean section. If you ask a woman about an operation, she asks ‘Could I survive without an operation despite the survival of the fetus?’ And if we say yes, she says, the child can die, I can have another child tomorrow and I don’t like to be operated on. So they don’t care about their child. All families, the father and the mother don’t focus on the child.”* **MCH focal person**

The mother discussed that she had learnt many lessons from her current pregnancy complications, in addition to this, she learnt a lot about the advantages of using a health facility for the benefit of the mother and her baby, including about family planning.

*“From this pregnancy I have learnt that going to the health facility is good for the health of the mother and baby….”* **The mother**

In addition, there was poor interaction among the hospital staff. For example, the senior midwifery nurses do not consult the junior physicians on the assumption that these junior GPs lack experience. Moreover, rather than teaching the junior GPs the senior physicians accept the existing trends of the midwives and as a result there was poor communication between the GPs and other senior staff.

*“The main thing is most GPs were new graduates and lack experience, second it is time consuming to make the decision. This system is well accepted by the seniors.”* **MCH focal person**

The main delay before reaching the hospital was lack of transportation followed by lack of awareness about the disease. For example, if a woman is convulsing, the family believes that she was attacked by an evil spirit rather than disease.

*“…Then they bring her to hospital after they failed many other trials. But lack of transportation is still a more severe problem and a mother will not die here if she is admitted early.”* **MCH focal person**

## Discussion and conclusions

This study found that previous pregnancy situation may repeat it self in the future pregnancies. Moreover, the health administrators use the ambulance for administrative purposes rather than for emergency health issues. On a positive note, attending a health facility is an opportunity for a mother to learn about existing health services in the facility such as family planning and the importance of ANC.

In the community, women who have had no obstetric problems during their previous pregnancies perceived that they will have no problems with their consecutive pregnancies and therefore feel that ANC and facility delivery was not useful. It was only necessary when severe complications occured that they attend health facilities. Moreover, women do not like to visit health facilities due to fear of pregnancy-related interventions like induction and instrumentation during delivery. Seizures due to eclampsia were perceived by the community as being caused by an evil spirit, and they prefer to take a preeclamptic/eclamptic mother to religious institutions rather than to health facilities. Women with preeclampsia/eclampsia may have underweight babies and visiting health facilities will be lifesaving for both the mother and her child.

Studies reported that not attending ANC, preeclampsia, multiple pregnancies, and bad obstetric history are significant maternal factors resulting in low birth weight babies and low birth weight babies are at a greater risk of having a disability and for diseases [17–19].

Preeclampsia/eclampsia is the result of ischemia in the tertiary villi of the placenta and affects all organs; the definitive treatment is termination of the pregnancy (removing the placenta). However, because of fear of instrumentation and the culture of being attended at home, the mother refused the procedure and went home to be attended at home; the labor spontaneously started at home after 2 days which may further affect the health of the mother and her baby.

Neonates delivered by multiparous women were at three-times greater risk of a low Apgar score compared with lower parity women, and both multiparity and low birth weight were independently associated with a low Apgar score (OR, 2.4) for low birth weight [20]. In this case, the mother is multiparous and her baby had a low birth weight and low Apgar score (6 at fifth minute). This shows that there is a need for further analytical research on the relationship between multiparity and low birth weight and low Apgar score.

An uneventful obstetric history often blinds multiparous women to possible dangers in childbirth, leads to low antenatal uptake, and late presentation to hospitals for obstetric complications. So robust patient education and use of accessible community health promotors like health extension workers (HEWs) and Ethiopia’s Women’s Development Army should be encouraged to improve antenatal coverage and early presentation to hospitals.

## Abbreviations

ANC: Antenatal care
AST: Aspartate aminotransferase
BP: Blood pressure
CBC: Complete blood count
C-section: Cesarean section
CTG: Cardiotocography
GP: General practitioner
HEWs: Health extension workers
ICU: Intensive Care Unit
IDIs: In-depth interviews
IRRB: Institutional Research Review Board
KIIs: Key informant interviews
LFT: Liver function test
MCH: Maternal and child health
n-TBA: Non-trained traditional birth attendant
PR: Pulse rate
RFT: Renal function test
RR: Respiratory rate
SPHMMC: Saint Paul’s Hospital Millennium Medical College
WHO: World Health Organization

## Authors’ information

The Author is an expert with different qualifications (MPH in Health services management, BSC in Public health, BSC in Computer science, Diploma in MLS) and is currently working as Head, Public Health and Health Policy, School of Public Health, Haramaya University, Ethiopia.
